# Annotations

**Published:** 1894-03-10

**Authors:** 


### Annotations.

**The Doctor or the Chemist?**

Mr. F. J. Bullar, M.A., F.R.C.S., in his annual address to the Southampton Medical Society the other day, put the saddle on the right horse when he said that it is ourselves, the practising members of the medical profession, who are responsible for the existence of the "prescribing chemist," and for other forms of quackery. "If the public are to be educated in these matters," he said, "it must be done by the medical profession, and we may well ask ourselves what the profession does to promote this public education, and whether we are not, after all, the persons to blame for the confusion of ideas as to the functions of chemist and doctor?" He tells us of a "lady who got out of a carriage" at a chemist's shop door and asked for medicine, because her "child had a cough." The chemist, of course, never even saw the child. Now in what sense are doctors to blame for the fact that they are classed by the public as a kind of superior chemists? Mr. Bullar tells us. We cut down our fees so low that we are obliged to see more patients than we can diagnose and treat properly, and so the public are not able to see a really solid and substantial difference between the mass of general practitioners and the well-dressed and exceedingly polite chemist. As a matter of fact, the chemist is often the more presentable and dignified man of the two, because he makes a better income, and does not ruin his manners and his temper by his slavish method of making it. Indeed, one does not quite see where the poorer general practitioner is drifting to. What with sixpenny consultations and twopence a quarter clubs, it is quite certain he cannot be honest. If all that a medical education can do for some men is to plunge them into poverty and knavery, it would certainly be better that they should save the cost of the education. We cannot help thinking that something might be done if the higher ranks of the profession would encourage in their hearts a little more comradeship for their sorely-bestead brethren in poor districts.

**"Selection" and Race Progress.**

The Milroy lectures, delivered before the Royal College of Physicians by Professor Haycraft, are of deep interest to those who hold that science should be put to the highest uses of which it is capable. Darwin has taught us the potency of the principle of "natural selection" on the large scale. Professor Haycraft thinks the time has arrived when the principle of "artificial selection" may be applied by peoples and nations for their own improvement and progress. Criminals, he says, marry and become the progenitors of other criminals; and he cites the well-known case of the Jukes family, which, through seven successive generations, "contributed to the community as their share of effort an unparalleled history of pauperism and crime." Why should we permit this kind of thing? asks Professor Haycraft. And the answer would certainly seem to be, because we have not yet sufficient intelligence and force of national character to prevent it. Professor Haycraft boldly proposes "segregation" as a remedy. The unredeemable pauper he would segregate, make him live in communities with others like himself, get as much work out of him as possible for his maintenance, put an absolute prohibition against his marrying, and in the course of two or three generations exterminate him, as we have exterminated wolves and dangerous snakes. The same course he proposes for the habitual criminal. The proposal is decidedly of the right order. It has our heartiest concurrence. On the other hand, he would encourage earlier marriages on the part of the mentally capable and morally sound, in order that they may become the progenitors of larger families. By these means he considers that the overwhelming residuum which makes life so grievously burdensome to the honest toilers would be diminished, and there would gradually be established a race of strong, capable, virtuous, and self-reliant persons. This prospect is also very agreeable; and if Professor Haycraft will now take some practical steps towards establishing some actual working methods of "selection," we shall, with the greatest possible enthusiasm, give him all the help in our power.

**Should Journalists Marry?**

Journalists, it is said, are to our science-loving generation what priests and teachers were to the generations before science was born. They are our prophets, our apostles, our teachers, our enthusiastic or judicial inspirers and exemplars. To men of this exalted calling, absolute independence of mind and clearness of judgment are indispensable. Can the journalist be clear in judgment and independent in mind if he "lives to write, and must write to live"? He may if he alone be dependent upon his writing; but when there is a wife at home, and, above all, children, whose shelter and table may be the price of a too transparent clearness of judgment and a too decided independence of mind, a new element enters into the calculation. Wherefore, it is urged, the journalist should not be exposed to these tremendous temptations to self-emasculation. At twenty he is a man. His mind is for the righting of hoary wrongs and for the building up of newer and nobler institutions. At forty he is—what? A writer—at least still a writer, but with his eyes "opened" we were going to say, but "closed" perhaps, and his backbone turned into a gelatinous notochord. It is a case of retrograde metamorphosis. Wherefore, it is said, he should not marry. He should be celibate like the clergy of the great Roman Church. Not marrying he need not write more than half or a third of what he now writes, and how much increased in might would be that one half! Moreover, he could write his own thoughts, and his own convictions, in his own form, and how much intensified in reality and power of self-propagation those thoughts and convictions would be! These be hard sayings for the journalist, and he will no doubt go on marrying. But if he would sacrifice himself on the altar of his epoch's progress and greatness, how swift might be the wings of that progress and how great might be that greatness! The journalist may ask the physiologist whether or not his health is likely to suffer if he remains celibate? Certainly not! The virgin man has precisely the same assurances of perfect health as the virgin woman.

